# A Multifunctional, Low Cost and Sustainable Neonatal Database System

**DOI:** 10.3390/children11020217

**Published:** 2024-02-08

**Authors:** Joaquim M. B. Pinheiro, Marilyn Fisher, Upender K. Munshi, Rubia Khalak, Kate A. Tauber, James J. Cummings, Jennifer B. Cerone, Meredith Monaco-Brown, Gina Geis, Rehman Chowdhry, Mary Fay, Anshu A. Paul, Carolyn Levine, Phillip Pan, Michael J. Horgan

**Affiliations:** 1 Division of Neonatology, Department of Pediatrics, Albany Medical College, Albany, NY 12208, USA; fisherm@amc.edu (M.F.); munshiu@amc.edu (U.K.M.); khalakr@amc.edu (R.K.); tauberk@amc.edu (K.A.T.); cumminj1@amc.edu (J.J.C.); ceronej@amc.edu (J.B.C.); monacom1@amc.edu (M.M.-B.); geisg@amc.edu (G.G.); chowdhr1@amc.edu (R.C.); faym@amc.edu (M.F.); paula5@amc.edu (A.A.P.); horganm@amc.edu (M.J.H.); 2 Neonatology, Ellis Hospital, Schenectady, NY 12308, USA; levinec@ellismedicine.org (C.L.); panp@ellismedicine.org (P.P.)

**Keywords:** databases, data quality, data systems, informatics, trends, quality improvement, research, NICU, neonatology

## Abstract

Continuous improvement in the clinical performance of neonatal intensive care units (NICU) depends on the use of locally relevant, reliable data. However, neonatal databases with these characteristics are typically unavailable in NICUs using paper-based records, while in those using electronic records, the inaccuracy of data and the inability to customize commercial data systems limit their usability for quality improvement or research purposes. We describe the characteristics and uses of a simple, neonatologist-centered data system that has been successfully maintained for 30 years, with minimal resources and serving multiple purposes, including quality improvement, administrative, research support and educational functions. Structurally, our system comprises customized paper and electronic components, while key functional aspects include the attending-based recording of diagnoses, integration into clinical workflows, multilevel data accuracy and validation checks, and periodic reporting on both data quality and NICU performance results. We provide examples of data validation methods and trends observed over three decades, and discuss essential elements for the successful implementation of this system. This database is reliable and easily maintained; it can be developed from simple paper-based forms or used to supplement the functionality and end-user customizability of existing electronic medical records. This system should be readily adaptable to NICUs in either high- or limited-resource environments.

## 1. Introduction

The rapid evolution of neonatal intensive care, despite limited good-quality evidence, results in a high degree of variation in care practices and outcomes, both across different neonatal intensive care units (NICU) and within a NICU over time [1,2]. The accurate quantification of key processes and outcomes of care in NICUs allows for continuous surveillance as well as comparisons among NICUs, making it possible to identify potentially better practices and benchmark results, which are essential elements of continuous quality improvement strategies [3,4,5,6,7,8]. Ideally, the data required for such quantification would be derived automatically from electronic medical records and administrative databases. However, administrative datasets are notoriously unreliable in capturing accurate diagnostic information [9,10,11,12], and they are generally not accessible to clinician decision makers [13]. Whereas electronic medical records have recently become commonplace in the United States and other high-income countries, they remain unavailable in most low/middle income settings [14]; furthermore, their reliability as a source of data for quality improvement or research purposes remains uncertain [10,11,15]. To address these limitations, some neonatal or perinatal databases are designed specifically for clinical surveillance, quality improvement and possibly research [1,16,17]; this allows for the a priori standardized definition of data elements, resulting in more reliable metrics than those obtainable from administrative or clinical databases. A reliable database is highly desirable to help guide neonatal care practices, but its feasibility is limited by the need for dedicated personnel and material resources to develop and maintain a complete, high-quality data set.

At Albany Medical Center, a pioneering perinatal database was developed [18], but it remained unused due to insufficient personnel resources to systematically abstract data from paper records. Following subsequent attempts at creating a sustainable database, we designed a neonatal data system which leveraged existing resources. We aimed to meet the needs for specific clinical, quality assurance, administrative, and educational functions of our NICU, while also providing data for participation in the Vermont–Oxford Network (VON) [1] and eventually supporting submissions to New York State’s Perinatal Data System (SPDS) [19]. Our simple, low resource, customizable data system has proven to be reliable and extremely useful, and has remained in continuous operation for over 30 years. Herein, we describe essential design features of our hybrid paper and electronic NICU database system, provide examples of processes that maximize data quality, and illustrate customized data elements which help us to gain a deeper understanding of neonatal diseases of local interest. Our experience in neonatologist-centered acquisition and use of neonatal data should prove useful to clinicians who wish to adapt some of these concepts to their own settings, whether their data sources are in electronic, paper or hybrid formats.

## 2. Materials and Methods

### 2.1. Structural Elements of the Data System

#### 2.1.1. Paper Form for Data Recording—Design and Deployment

Paper forms have unique affordances, including instantaneous access that allows for the very rapid checking or circling of standard items, corrections that remain visible, and the ability to add free text, draw arrows between fields, or leave notes or questions for review before data entry into the database [20]. To maximize the feasibility of use among neonatologists, we decided that each patient’s form should be restricted to a single paper sheet, with information on the front and back (Supplementary Materials S1). On admission to the NICU, demographic and perinatal information is recorded on the top half of the first page by the admitting neonatologist, whose initials are then circled; on the bottom half of this page, discharge information will be recorded by the discharging neonatologist. Information in the boxes with smaller font is entered only for neonates eligible for submission to the VON Very Low Birth Weight (VLBW) database and is thus left blank for most admissions. On the second page, standard diagnoses and interventions that accrue during the NICU course are organized by major systems; “Other” common diagnoses are aggregated in a larger category towards the bottom. Within each system, a free text field allows for additional entries or details. A section for quality assurance or patient safety items appears at the bottom, along with an area for notes to self or others, and a list of studies that are circled when the infant is enrolled.

For immediate access, the form was placed at the end of the bedside chart binder, so that the second page faced the (blue) billing form, required to be filled out by the neonatologist during daily rounds. This arrangement ensured that when the attending neonatologists opened the chart to enter the billing codes on the right, they would see the facing page displaying the data sheet, allowing for the documentation of any new diagnoses, interventions or other significant events in a matter of seconds, during routine workflow. In addition, this sheet provides an instant listing of key diagnoses and events during hospitalization, which serves as a quick, structured visual summary and aids in communication. This feature is useful for neonatologists, but also for Pediatric Ophthalmologists, who quickly review the form to obtain basic information before screening exams for retinopathy of prematurity, and then enter the results of their exam directly onto the form. The data forms were on white paper in the first year, but after several were lost when charts were disassembled upon discharge, they have been printed on light pink paper. The distinctive “pink sheets” are easy to see and virtually never lost. With the recent switch to electronic clinical documentation and billing, pink sheets for patients on each NICU team have been moved to a binder that is carried by the on-service neonatologists, who variably updates the sheets on rounds (with the binder on the mobile computer cart), during daily sign-outs or while undertaking electronic billing. The contents of the sheets have been repeatedly modified to meet the requirements in reporting to VON, and to accommodate local needs.

#### 2.1.2. Electronic Database Structure and Basic Maintenance

The original electronic component of the database system was built as a spreadsheet in Lotus 123 for DOS, and eventually translated into a multi-worksheet Excel^®^ 365 workbook in XLSM format (Microsoft Inc., Redmond, WA, USA). Macros have been used from the outset, to automate and accelerate repetitive functions, from data entry to report generation. With the advent of HIPAA (Health Insurance Portability and Accountability Act) regulations, password-protected data files for annual admission cohorts are located in a hidden folder on a server of the institutional network, with automated back-up and behind a firewall. Folder visibility and file access are restricted to the administrative assistant, NICU data registrar, and database administrator; any new user in these capacities must have access approved by a Department of Pediatrics administrator. Data are entered by an administrative assistant in the Neonatology office for all infants admitted or discharged during the previous calendar day. A new record is added for each NICU admission or delivery room neonatal death, through a macro that creates default values for each column; only a few elements with demographic data are entered on admission. For discharged patients, data entered on their individual “pink sheet” replace the default values. Admission information is derived from the NICU daily admission log generated by a receptionist in the NICU. Pink sheets are removed from each patient’s chart upon discharge from the NICU and provided to the Neonatology administrative assistant, along with the daily discharge log. At this stage of data entry into the spreadsheet, pink sheets that are missing, incomplete or contain implausible information are returned to the discharging neonatologist and marked temporarily as incomplete in the database.

Our NICU has approximately 800 annual admissions, thus averaging 2 to 3 daily admissions and discharges. The basic steps described above can be accomplished by the NICU administrative assistant in under 4 min per day, based on time tracking performed in the early years of implementation of our system. This work translates to 0.012 full time equivalents, but considerably more time may be required to follow up when sheets are incomplete, as described below.

### 2.2. Database Contents

The paper forms contain all the information necessary to submit to the VON VLBW database [2]. Details such as the location of a diagnosis such as necrotizing enterocolitis (NEC) can be indicated by marking the adjacent checkbox (at our center), or the circle “O”, for “outside hospital”. These features allow us to continue using a single sheet, despite the increasing complexity of VON data forms over the years. We use the VON definitions for diagnoses and interventions, which are almost entirely harmonized with the NICU module of New York’s SPDS [19].

Since most infants admitted are not of VLBW and thus not reported to the VON VLBW database, there are additional neonatal diagnoses and interventions, including common ones that can simply be circled, while others can be entered as free text. The same applies to quality/safety incidents, which can be entered as text, or by filling in a short prompt on items of current focus, such as the date of an unplanned extubation event, or the volume (mL) of surfactant at which a specifically defined airway obstruction event occurred. This approach enables attending neonatologists to record standardized information in a matter of seconds, during routine workflow.

Some diagnoses have been defined and standardized locally, to minimize variations in terminology and conceptualization among neonatologists when there is no external consensus on such items. For example, we defined “presumed sepsis” as culture-negative sepsis that is treated or intended to be treated; we applied the standard categorization of early- and late-onset sepsis to this diagnosis. We defined “NEC-like disease” as a condition where a primary intestinal disorder fails to meet standard criteria for NEC, but there are persistent features of bowel ischemia or other dysfunctions which prompt clinicians to treat for a full course of bowel rest and antibiotics. We apply the “BPD” diagnosis to infants who meet standard BPD criteria and also to those who do not meet such criteria but are being maintained on diuretics for chronic lung disease. We also apply consistent criteria for diagnoses, e.g., hyponatremia (“LoNa < 130”) and direct hyperbilirubinemia “cholestasis > 2 mg/dL” (Supplementary Materials S1)

The core data for all infants are entered into the electronic spreadsheet by an administrative assistant. For VON-eligible infants, some VON-specific data elements (e.g., caffeine, admission temperature, discharge weight and head circumference) are left out of our local database but entered only by the NICU data registrar into VON’s eNICQ application. In this manner, we avoid a duplication of efforts between the Neonatology administrative assistant and the NICU data registrar, while benefiting from participation in VON and New York SPDS and enabling our own customized data analyses.

### 2.3. Processes to Ensure Data Quality

Multiple processes are applied at various steps of data acquisition and analysis, to ensure completion and maximize the accuracy of the data (Supplementary Materials S2). An important feature of our database is that the attending neonatologists, who are most qualified to make accurate diagnoses, directly record the information on the pink sheets. To this end, we orient new neonatologists to the pink sheet contents and best practice workflows. Some data elements on the forms have associated cutoff values, which helps standardize the definitions, as noted previously. During pink sheet completion, systems without relevant diagnoses or interventions should have “0” checked (this could take the form of a large oval encompassing the 0 of several consecutive rows, as exemplified in Supplementary Materials S1). Systems left blank are highlighted in yellow by the administrative assistant, who flags the record as incomplete in the database and returns the sheet to the discharging neonatologist for completion.

Upon data entry into Excel^®^, the worksheet has prompts for data completion, e.g., using conditional formatting to colorize cells specific to babies eligible for the VON database, such as antenatal steroids, timing of the surfactant, ROP exam. We use data validation features to restrict medical record numbers to valid formats, dates from the present to 360 days prior, and plausible ranges for variables such as birth weight (grams) and gestational age (weeks). Logic checks between separate variables are used to alert for probable data errors. For example, gestational age is entered on admission and a redundant GA variable (weeks and days) is entered after discharge, in the discharge area of the worksheet; incongruence between these spatially and temporally separate values triggers an error alert. A similar approach is used to detect discrepancies between ventilation and oxygen use, date off oxygen and BPD diagnosis, and date of admission preceding date of birth, among others. After discharge data have been entered, the record shows the sum of likely errors, while a header cell shows the total of such errors in the worksheet. Likely errors are highlighted in the pink sheet or noted on a sticky note and returned to the discharging neonatologist for correction or verification. Following these steps, all pink sheets for VON-eligible infants and all infants who died, plus those with particularly complex records, are reviewed by the database manager (JP), who verifies key information and scans the worksheet for obvious data entry errors. At this step, discharge data are cross-checked against a small database of key events on approximately 10% of the infants, which is gathered in parallel through a process affectionately named “The Pink Sheet Police”, in which the database manager aggregates data items submitted by selected neonatologists, most often derived from sign-out or similar discussions. An annual review by the neonatology group of the frequency and types of errors detected through this system allows the retrospective correction of key omissions, and it also aims to prevent recurrent errors incurred through individual or group habits. This feature helps to maintain a common understanding of data item definitions and encourages a culture of data accuracy among the neonatologists.

Additional cross-checks are performed when there is an opportunity to compare selected data with those obtained independently from other sources, such as administrative, QI or research datasets. Whether performed contemporaneously with data collection or retrospectively, even years later, these routine exercises have proven useful for recurrently assessing the validity of our data with respect to specific variables.

To ensure that all eligible records are entered in the database, even for liveborn neonates of any gestational age who die in the delivery room and who may not have been attended by a NICU team, the hospital’s birth certificate/vital records office provides the database manager and NICU administrative assistant with notifications of all neonatal deaths in the hospital, irrespective of location.

Finally, during data analyses for annual reports, simple data cleaning methods [21,22,23], along with deliberately performing a few of the analyses manually while directly visualizing the records, allows for the detection and correction of additional errors and error patterns that would likely go unnoticed if all analyses were automated.

### 2.4. Data Analysis and Reporting

#### 2.4.1. Continuous Reporting

For administrative and patient management purposes, the database includes individual worksheets with current year-to-date daily and monthly census graphs (Supplemental Figure S1). Other worksheets use customizable macros to output lists of current inpatients sorted by length of stay, incomplete records by discharging attending, monthly mortality and morbidity/QI events (along with autopsies and diagnoses pending at discharge), deaths in the current year, monthly VON-eligible admissions, weekly candidates for retinopathy screening, ROP eligibility, surgical discharges and discharges in the last 10 days. These reports facilitate various administrative, patient care and quality improvement functions. Some reports were used transiently for some years, for example, hearing screen results, and reports for hearing screens that were missed or were referred for outpatient follow-up by the Hearing Center; this feature ensured full compliance during the initial implementation of universal hearing screening.

#### 2.4.2. Annual Analyses and Reporting

Datasets of annual NICU service admission cohorts are analyzed for patient characteristics, along with major interventions and outcomes, in conjunction with analyses of reports from the VON VLBW database. Quantitative analyses are mostly descriptive, stratified by population subgroups of interest. We supplement VON reports with analyses of related outcomes, e.g., presumed sepsis relative to culture-positive sepsis, sepsis rates in VLBW versus larger neonates, and NEC-like disease in parallel with NEC, among others. We also graph annual values in the context of long-term trends in patient characteristics, interventions and outcomes. Survival in very preterm neonates by gestational week is reported annually and aggregated over the past 5 years to provide more stable estimates used in perinatal counseling and decision-making. No other risk-adjustment procedures are carried out routinely. Most routine analyses are performed using custom-built worksheets in Excel^®^ 365, while some supplementary analyses have used one of several software applications with statistical process control charting capabilities. Trend graph examples in this manuscript were generated through Stata 17 scripts (Austin, TX, USA). Summary reports are shared with Neonatology, Obstetrics and Quality Management staff, and there is an extensive annual presentation of the results with group discussion.

A qualitative, thematic analysis of incidents entered as free text in the Quality Assurance/Morbidities box is performed monthly and discussed at the Morbidity and Mortality/QI conference [24].

Finally, the results of annual admission cohort data analyses are compared with analyses of similar data from alternate sources (e.g., selected audits, QI projects, research studies), when available. Results from VON reports on items such as intraventricular hemorrhage, steroids for BPD, and patent ductus arteriosus (PDA) ligation are routinely compared with results of automated analyses from our raw data, using a customized Stata code. This allows us to assess the reliability of data collected through this system, and to correct errors revealed by inconsistent results.

For data security reasons, reports for aggregate data needed in preparation for designing research projects are routed through the database manager. Although the original database use was determined by the Institutional Review Board (IRB) to constitute quality assurance and not research, clinicians requesting reports that may be used for research purposes must first obtain IRB approval or exemption. A record of such reports and of specific patient lists provided to researchers after approval is kept by the database manager.

## 3. Results

The database system has been in continuous use for over 30 years, without material or personnel resources being allocated specifically for this system. The following examples highlight key functional processes and results derived from this database. During 1993–2022, there were 22,728 infants admitted to the Neonatology service, of which 4224 were VLBW (birth weight < 1501 g), and 65.1% inborn. There were 402 delivery room deaths among all live-born infants, including those of GA < 20 weeks.

The continual preoccupation with data quality has given us an understanding of which variables contain accurate information, while also allowing us to correct errors in either of the source data sets. For example, a report on 241 PDA ligations through 2014 revealed a single missing case in the database, which was identified in one of the alternate sources of partial data, yielding a sensitivity of 99.6%. In 2022, with a new categorization of PDA surgery in the VON database and a new NICU registrar entering data, we found an excess of PDA closures by interventional catheterization in the VON report compared with our local analyses, which were verified to be correct; this allowed us to correct the VON data and re-educate the registrar, who had misunderstood the coding of this key variable, even while coding other variables correctly.

The annual “Pink Sheet Police” cross-checks, focusing on data items that are either important or most likely to be missed, typically reveal proportions of about 15% inaccuracies, on items such as date off oxygen or omission of some transient mode of respiratory support during weaning (e.g., nasal CPAP used between non-invasive positive pressure ventilation and high-flow nasal cannula). However, a few instances of postnatal steroid use, nitric oxide administration, bowel reanastomosis surgery and even sepsis have been missed, which helps us to identify patterns leading to inaccuracy and re-emphasize strategies for timely and accurate data entry on the pink sheets.

**Figure 1:**
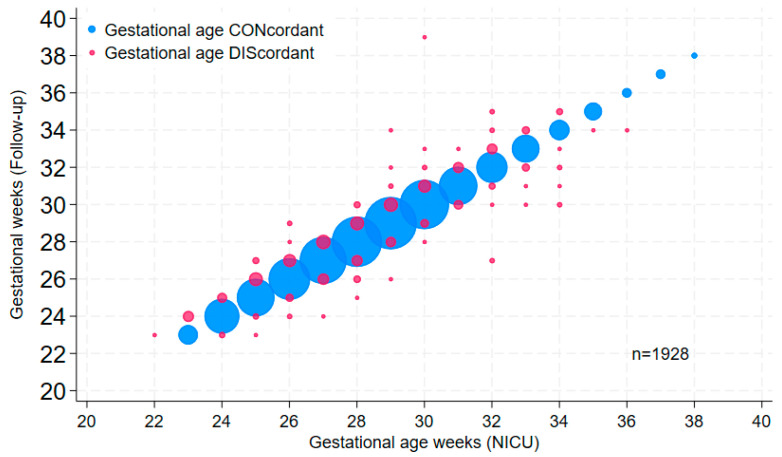
Bubble plot of gestational age values in the NICU database versus corresponding values in the 1993–2006 cohort of the high-risk follow-up database (Follow-up). Bubble size is proportional to the number of patients in the data point; pink bubbles are discordant values.

Because accurate gestational age recording is critically important, we augmented initial random audits of a small number of records with systematic analyses of larger cohorts, such as annual datasets from different sources. We found that the most efficient and useful method was the graphing of gestational ages from the same patients in two distinct datasets; if gestational data from the same patients (using a common identifier) are entirely concordant, the dot plot (or bubble plot, as in Figure 1) will yield a perfectly straight line; deviations from the line of identity indicate cases in which at least one of the gestational ages is incorrect. Comparing data derived from delivery room records, NICU log entries, admission notes and discharge summaries to pink sheet data allowed us to identify, understand and mitigate GA inaccuracies in the early years of the database. Interestingly, when using this approach to compare GA in the NICU dataset with GA in the outpatient neurodevelopmental follow-up dataset containing more than 1000 high-risk neonates referred over 14 years, a clinically important frequency of discrepancies was apparent (Figure 1). Further inquiry into the discrepancy patterns revealed that the follow-up database relied on a variety of GA data sources (e.g., admission information in logs or chart notes, consultation notes, discharge notes); none of these was systematically verified.

**Figure 2:**
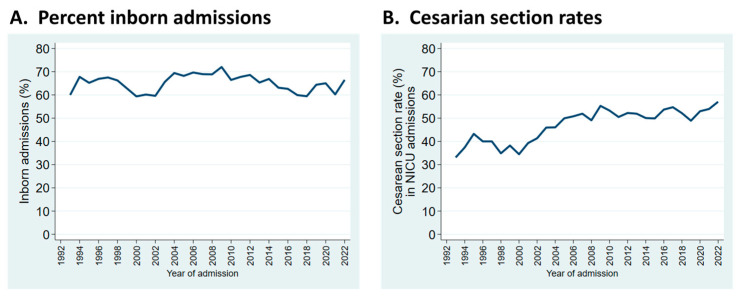
Trends in patient characteristics by year of admission to the NICU. (**A**): Admissions who were inborn; (**B**): rates of cesarean section birth in infants admitted to the NICU.

Trends observed in selected patient characteristics and outcomes over the years reveal significant changes that reflect external influences and changes in practice. For example, the proportion of infants who were inborn reflects referral pattern changes with healthcare reorganization in our region but is relatively stable in the long term (Figure 2A). The sustained increase in cesarean section rates reflects obstetrical practice (Figure 2B).

**Figure 3:**
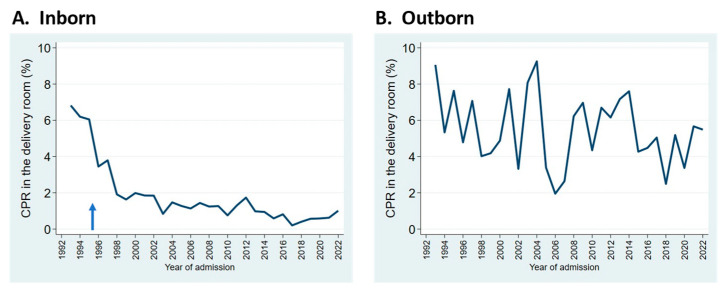
Trends in the rates of cardiopulmonary resuscitation (CPR) in the delivery room for inborn (**A**) and outborn (**B**) neonates admitted to the NICU. Arrow indicates start of overnight in-hospital neonatologist coverage.

Resuscitation practices in the delivery room have evolved noticeably. The frequency of delivery room cardiopulmonary resuscitation (DR CPR, comprising chest compressions and/or epinephrine administration) decreased dramatically in the mid-1990s at our center, coinciding with the practice of having attending neonatologists remain at the hospital overnight, starting in late 1995 (Figure 3A). For outborn infants, rates of DR CPR have remained higher, and more variable (Figure 3B).

**Figure 4:**
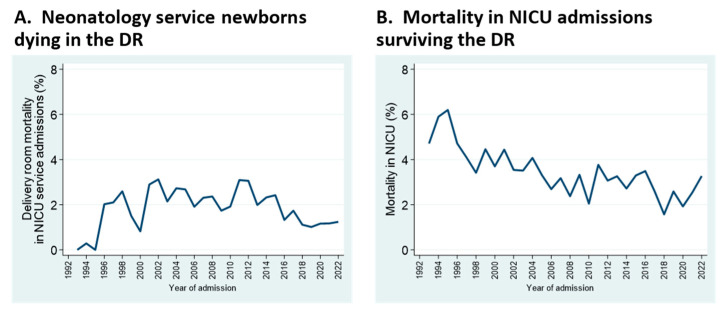
Mortality trends for deaths occurring within the delivery room (**A**), and in NICU proper (**B**). See text for details.

Mortality patterns have changed over the years, as well. Neonatal deaths in the delivery room, which include live births of any gestational age, increased and more recently decreased; this mostly reflects changes in perinatal practice at borderline viability (Figure 4A). Overall mortality in NICU admissions has decreased gradually, despite increases in admissions of neonates at the edge of viability (Figure 4B). Of note, the denominator for delivery room (DR) mortality is total admissions to the Neonatology service (i.e., NICU admissions + DR deaths), whereas the denominator for in-NICU mortality comprises admissions to the NICU proper, whether inborn or transported from referring hospitals.

**Figure 5:**
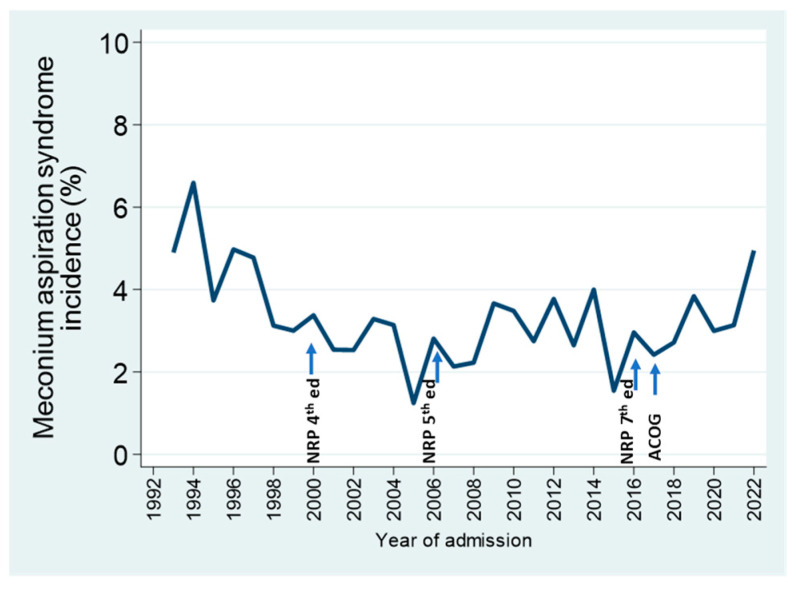
Percentage of annual NICU admissions of GA > 34 weeks diagnosed with meconium aspiration syndrome in relation to recommendations for the intrapartum and neonatal management of infants with meconium-stained amniotic fluid in various Neonatal Resuscitation Program (NRP) editions, and in the statement by the American College of Obstetricians and Gynecologists (ACOG).

The management of meconium-stained amniotic fluid has evolved over the years, in response to changes in national guidelines for pediatric and obstetric practice [25,26], reflected in successive editions of the Neonatal Resuscitation Program [27]. As routine suctioning interventions were gradually abolished from both neonatal and intrapartum obstetrical management, the proportion of admissions with meconium aspiration syndrome did not increase; rather, it decreased during the first decade, and rates remained stable over the next 15 years; it is too early to tell whether the last 5 years indicate an upward trend in the incidence of this diagnosis (Figure 5).

**Figure 6:**
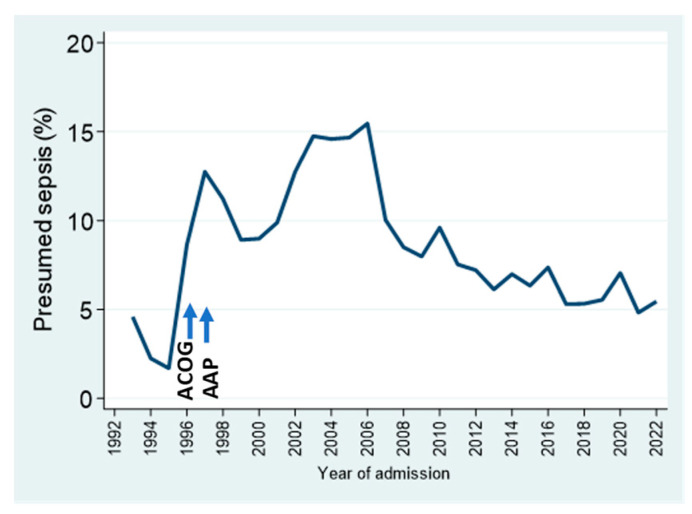
Presumed (culture-negative) sepsis trends by year, as a percentage of NICU admissions. See text for details.

Presumed sepsis (culture-negative sepsis that was treated fully or intended to be treated) is one of the locally defined outcomes tracked by our database. The prevalence of this diagnosis increased sharply with the widespread implementation of group B streptococcal intrapartum prophylaxis in the mid-late 1990s, coinciding with recommendations by the American College of Obstetricians and Gynecologists (ACOG) in 1996 [25] and by the American Academy of Pediatrics (AAP) in 1997 [26]. The subsequent focus on antimicrobial stewardship, plus guidelines promoting alternate approaches to the diagnosis of early-onset sepsis, have been associated with the gradual decrease in this measure over the last 15 years (Figure 6).

Starting in 2000, we added the box for the rapid recording of quality or safety-type incidents on the pink sheets. This yielded 3809 incidents recorded in 2646 infants (14.8% of the admissions). On average, 14 events per month undergo thematic analysis and are reviewed at the monthly Neonatology Morbidity and Mortality/QI conference. Themes under special focus result in the temporary introduction of prompts to facilitate event entry, e.g., “Unplanned extubation date(s) ________” on the current form (Supplementary Materials S1). Even with the more recent establishment of a hospital-wide electronic incident reporting system, entries by physicians on the pink sheet forms remain more frequent than those reported through the electronic system. We have not formally compared patterns of incidents reported through the two systems.

## 4. Discussion

In this manuscript, we describe the development and illustrate selected uses of a dependable, enduring local NICU database that is highly customizable and multifunctional. It provides support for clinical care, administrative functions, quality improvement tasks, education and data analyses preparatory to research. This data system required no specific funding, utilizing only existing human and basic material resources and integrating into existing workflows. This makes the system time- and cost-efficient, while having frontline neonatologists record data on diagnoses improves its reliability. These characteristics should make the implementation of this database system feasible in a variety of environments, whether clinical records rely primarily on paper charts or on electronic health records (EHR) [28]. It can possibly be adapted to a country with limited resources.

This system takes advantage of the affordances of a paper form [20,28] to allow quick, convenient and immediate documentation. This minimizes time required for the task while maximizing accuracy, as neonatologists record diagnoses and interventions when events occur, without the need for retrospective recall and review. We assume that the neonatologist who confirms a standardized diagnosis while attending to the infant is more likely to record this information accurately than the physician who discharges the infant 3 months later; this neonatologist is uniquely qualified for this task, relative to a research or clerical staff member uninvolved in the patient’s care and relying on narrative medical record documentation. This may be a key factor in determining data quality. Conversely, as additional date fields were added, the original date items (final date on oxygen and date of final extubation) became less reliable in neonatologists’ entries on the pink sheets, as there may be multiple attempts at weaning off those modes of support, which increases the data task burden. The introduction of high (air) flow nasal cannula has contributed to errors in the date off oxygen. For purposes where highly accurate data on the time of oxygen removal are essential, cross verification against the electronic medical record or other reliable data source should be performed.

### 4.1. Required and Suggested Resources

The low cost of materials needed to develop this system is explained by the need for light-colored paper sheets, since a computer running Excel^®^ or a similar spreadsheet or database program is already available in most settings. For our NICU, the estimated cost of 800 printed pink sheets per year, including toner, is about USD 58. Essential personnel beyond the frontline clinicians completing the forms includes, at a minimum, an administrative assistant for data entry and a neonatologist capable of performing analyses and database maintenance. A list of resources and basic functionalities needed to implement this system or similar alternative arrangements is presented in Supplementary Materials S2, along with comments that supplement the details in Methods.

### 4.2. Data Quality Considerations

Ensuring data quality is critical, whether large or small datasets are analyzed. Analyses based on large numbers will often produce statistically significant results, which may be meaningless in practice, or even misleading. When unexpected or highly improbable results are obtained, verifying the veracity of the underlying data should be the first priority [29,30]. Because plausible results may be derived from erroneous data, plausibility is an insufficient criterion for data quality. Thus, accuracy should be systematically verified. Data quality can be maximized through the design of the data acquisition process, using both electronic and manual methods to facilitate and prompt accurate data recording, by resorting to a system of random and non-random audits [31]; allowing feedback to originators of the data; using the most reliable sources of information (in our case, the attending physicians, for data on diagnoses) at one or more of the data acquisition steps; and habitual cross-validation with other datasets at every opportunity. Additional steps of data cleaning and validation should be applied before any analyses are undertaken [10,22,32].

Gestational age is the single most important data element on a neonatal record. It underlies decisions of direct consequence to individual neonates and their families, such as resuscitation at the edge of viability, outcome prediction, eligibility for screening and treatment protocols and the timing of various interventions. Analyzing outcomes such as survival from datasets that contain inaccurate GA can yield erroneous results which may impair clinicians’ and families’ abilities to engage in collaborative, ethical decision-making [33,34]. Yet, discrepancies in estimated GA within a prenatal record, and errors in its transfer to the corresponding postnatal record, are common [35]. Even within an electronic medical record for a single neonate, gestational age values appearing in different locations or data elements of the record can be discrepant [32]. Our own experience with various paper and electronic obstetrical data sources corroborates these reports. We have not assumed that GA derived from obstetrical systems, whether manually or by the electronic auto-population of neonatal records, is accurate. An examination of error patterns in dot plots of GA from different data sources, along with process mapping to characterize the paths of GA values and the most common sources of GA error, led to the targeted education of prenatal and neonatal care providers, and to the creation of independent admissions and discharge GA data elements in our database, with built-in alerts for discrepancies. Nevertheless, this did not prevent discrepancies in GA values between the NICU database and the follow-up database, which obtained its data from various available sources without cross-validation. After analyses revealed considerable discrepancies in GA values (Figure 1), the follow-up database team was prompted to derive its GA data from discharge summaries. A similar strategy, comparing database data with overlapping data in VON datasets, or in various other clinical or QI datasets, was applied to examine the accuracy of data on admission temperature, antenatal steroids, postnatal steroids and birth weight, among other factors. Although we perform these and previously mentioned comparisons frequently to maintain and improve data quality, we have not formally sought to quantify error rates in this or other datasets; typically, we find that for most variables, data in our system contain fewer inaccuracies than those from other data sources. By comparison, a system in the UK relying on admission data entry by junior doctors had significant initial rates of incompletion and inaccuracy [36].

### 4.3. Uses of the Pink Sheet/Database System

Our hybrid database system has multiple uses. For immediate patient care, the pink sheet is used by neonatologists and ophthalmologists as a convenient, at-a-glance summary of an infant’s main problems and treatments. We also use it as a rapid communicator of initial UVC position and adjustment (e.g., high → OK, Supplementary Materials S1, p. 4); these and other occasional reminder notes are not entered in the database. The database generates a list of patients eligible for retinopathy screening. It has aided in effectively implementing universal hearing screening by identifying unscreened neonates. Additionally, we use the database to generate a monthly report associating the discharging neonatologist with diagnostic testing pending at discharge and needing follow-up, such as genetic tests, autopsy, or other pathology reports. These reports are reviewed at monthly mortality/morbidity/QI conferences, and the follow-up information is invaluable for practice-based learning for both neonatology staff and trainees, while it is also useful to guide follow-up with families and community pediatricians.

Aside from practice-based learning, a database such as ours is also a requirement by the Accreditation Council for Graduate Medical Education for training neonatology fellows. The ability to perform timely, targeted analyses of local data, in addition to systematically tracking locally relevant outcomes and interventions, promotes the habit of evidence-based quality improvement. Routine communication and discussions of results among faculty, trainees, mid-level providers and other NICU staff creates shared understanding, which supports consensus in changing care practices. The system for the quick annotation of safety incidents or quality concerns at the bedside is time-efficient. As these events are reviewed and discussed by fellows at the monthly QI conference, alongside events reported in the hospital’s electronic reporting system by any of the NICU staff, the process is valuable for both clinical QI and education.

We also use the system for administrative functions, including tracking and graphing the NICU census by day and by month. In addition, a quarterly report is issued to the Departmental Business Office, as this database was identified as the most accurate source of NICU admission counts to support state-sponsored high-risk follow-up grants to the Children’s Hospital.

The dominant function of the database system is to characterize practice trends and support locally driven quality improvement, as illustrated through selected outcomes shown here. It also supports collaborative QI through VON membership, in addition to quality assurance mandates such as New York SPDS. Either directly or through VON, it can provide ad hoc data needed for external reporting to national agencies or consortia such as the Leapfrog Group.

Another function of the database is to generate reports through customized queries, in preparation for potential research by faculty, trainees or other staff. Reports obtained through the data manager contain estimates of population demographics, or numbers of patients with specific conditions or interventions; lists with identifiable patient information require prior IRB approval.

Finally, the system is highly customizable. In addition to the presumed sepsis diagnosis detailed above, other locally developed items include NEC-like disease, airway obstruction events during surfactant administration, enrollment in clinical trials and nasal trauma, among others. These items can be added temporarily, as was the case with language spoken at home, which we tried for 1 year.

### 4.4. Limitations

There are obvious limitations to this report, primarily related to the fact that the processes described herein were implemented successfully at only one institution. Because the specific components of the system were developed to integrate resources and workflows at our institution, it is not a turnkey system that can be instantly implemented elsewhere without modification. Nevertheless, all the functional elements of this system are widely available, without the need for extraordinary physical or personnel resources or significant investment; selected elements must be adapted in a modular fashion to other settings. We have not estimated the marginal time commitment by neonatologists to update pink sheets, but it is minimal, at most. Likewise, the time required for database administration and data analysis has not been estimated, and these functions have been assumed within clinical and academic work allocations, without specific protected time or compensation; it is unclear whether this would be easy to replicate in other settings. Furthermore, the efficiency and depth of understanding associated with assigning system administration and data analysis functions to a single clinician may be diminished if those functions are distributed to distinct individuals, especially if they are not clinicians.

Another limitation of the system is the deliberate restriction to two pages, which constrains the number of items that can be functionally arranged on the sheets. In any case, the system was deemed quite useful in its original form, in which the sheets contained less than half of the current items. To the extent that this system may duplicate other data systems existing in our setting, it can potentially contribute to a wastage of personnel resources [16,28], but no other system, nor combination of systems, can reliably perform most of its functions. However, as we introduce other electronic data systems and change workflows, we will need to remove unnecessary duplication, while maintaining strategic redundancies that will help to assess the quality of data across all sources.

We have not fully exploited the potential of this data system. For example, while we perform routine thematic, qualitative analysis on quality/safety incidents at monthly mortality and morbidity/QI conferences [24], we have not examined the relationship of items recorded on the pink sheets to those submitted through the institution’s electronic incident reporting system. Also, we have not performed a formal study of data quality metrics on most variables. The educational value of the system could be enhanced by increasing the role of the fellows in the routine updating of the sheets, although this might diminish data quality and erode the attending neonatologists’ habit of consistent pink sheet access and completion. While we have used the database to support numerous quality improvement and research projects, it is not immediately available to all neonatologists and other NICU staff, due to data security constraints. The current Excel^®^ 365 files cannot track access by users, and they contain some quality assurance information, in addition to clinical data. Thus, use of the data sets must be secured through physical and electronic means, and additionally controlled by a manager to ensure that appropriate, limited access conforms to federal rules and local IRB regulations. Switching to a more sophisticated software platform might improve access while maintaining data security, but this would require significant time, cost, and personnel expertise. Examples of neonatal data systems integrated with EHR include BadgerNet in the UK’s National Health System [37] and BabySteps data warehouse [38] from a large, private, multicenter neonatology group in the US. Both these systems require enormous resources for development and maintenance, which are not possible in many centers; also, they are not readily customizable based on user needs.

## 5. Conclusions

We have demonstrated the feasibility of creating and sustaining a uniquely neonatologist-centered NICU database that is reliable, multifunctional, time-efficient and highly customizable. Because this was achieved without specific funding, utilizing only existing human and material resources, it can be adapted to a variety of settings, using a locally designed combination of paper and common computer programs, aligned with available staff and their respective workflows. We speculate that essential ingredients in the successful maintenance of our system include the reliance on neonatologists for data collection and their involvement in analyses, the gentle but constant attention to data quality, the expectation that results are routinely used to support collective decision making, and the provision of basic information that enables the development of projects initiated by individuals or small groups. These features have helped to generate a culture among attending neonatologists in which confidence in the database reports and their usefulness to individuals and the group encourages continued contribution to this collective resource.

## Data Availability

Restrictions apply to the availability of these data. Data were obtained from AMC NICU databases and are available from the authors with permission from the AMC IRB. A sample Excel^®^ 365 workbook in XLSM format, containing simulated data and built-in macros, will be made available “as is” from the corresponding author upon written request. Full functionality requires that macros are enabled in the user’s Excel^®^ account, which may require approval from the local information technology security officers.

## Supplementary Materials

The following supporting information can be downloaded at: https://www.mdpi.com/article/10.3390/children11020217/s1, Figure S1: Automated graph displaying NICU daily census, and average for the year-to-date census. Supplementary Materials S1: Pink Sheet forms version 2022, completed with simulated data. The sheet for Baby Doe (pages 1 and 2) exemplifies items completed for a very low birthweight infant, including all of those required for the VON VLBW database, in addition to data entered in our local database. The sheet for Baby Moe (pages 3 and 4) was completed for a term neonate, whose data are entered only in our local database. Supplementary Materials S2: Resources and basic functions needed for the implementation of the database system.
