# Supplementary material for: A Multifunctional, Low Cost and Sustainable Neonatal Database System
Source: Children (Basel). 2024 Feb 8;11(2):217. doi: 10.3390/children11020217 (PMC10887617; doi:10.3390/children11020217)

Supplementary Materials S1, p1-4

2023 Albany Medical Center, NICU QI Database 2023

(Place Label here, or write)

Last Name: MOE

Sex: ✓ M __F __U *If multiple birth:* A B C D

Medical record #: 8888888

MOE Male

Date of: Birth (MM/DD/YY): 07/15/23

Admission Date: ___/___/___

Ethnicity+ Race (mother): __Hispanic? + __Black ✓White __Asian __Native American __Pac Isl __Other

Birth Location: ☑Inborn ☐Outborn at ______________________ *Hospital* +(VON) **AMC adm for:** __Med Rx/Diagnostics __CoolingRx __Surgery __Chronic Care __Growth/DC planning __Other

Birthweight: 4125 *grams* *Gestational age:* 38 *weeks* *Apgars* 1 / 5 5 (7 10)
(=9999 for NICU readmissions; complete only first 3 lines above)

⇨ ☐ Admitting Attending (circle): JBC RC MFay MF GG MH RK MMB UM (JP) KT

****

ADMITTING ATTENDING VERIFY ABOVE & COMPLETE ADMISSION SECTION:

Delivery: ☐*Vaginal* ☑*C/Section*

⇨ **0 DR INTERVENTION(s):** ☑O2 ☑CPAP ☑Bag-mask/PPV ☐NasalIPPV ☐LMA ☐ETT(any) ☐Epi ☐Compressions

If ≤ 1500 grams *OR* ≤ 29 6/7 weeks, liveborn (VON criteria), enter these 9 items:

Prenatal Care (1 office visit): ☐Yes / No ☐ **DM**$_{any}$ Yes / No ☐ **Chorioamnionitis** Yes / No ☐ **Hypertension (any)** Yes / No ☐ **MagSO4** Yes / No

☐ **Best EGA** ____ weeks ___ **days** **Antenatal steroids:** __☐None ☐Incomplete ☐Complete (>1 - <7 days)

☐Birth **Head Circ** ____.___cm ☐ **First AMC Temperature** ____.___ºC ☐Within 1 hour ☐At >1 hour of life

************************* DISPOSITION / DISCHARGE *************************

Chart to: Cerone Chowdhry Fay Fisher Geis Horgan Khalak (Monaco-Brown) Munshi Pinheiro Tauber

If died, *Date: (MM/DD/YY):* ___/___/___ **DR death?** _0_ /☐Yes **Autopsy** _0_ /☐Yes **inNICU<12 hrs?** _0_ /☐Yes
(*If DR death, note also: Surfactant, Malformations, on reverse side*)

If discharged, *date: (MM/DD/YY):* 7/23/23

To: ☑*Home (or foster care)* ☐*E6 Nursery/C7/D7* ☐*PICU* ☐*Other Hospital/(Name)* ______________________

ALL DIAGNOSES & RXs FINALIZED? Check small print items **& boxes for VLBW/VON babies…**

If ≤1500 g BW ***or*** ≤29 weeks (VON): Date when 36 wks PMA: ___/___/___ (N/A if D/Cd)

1. **Respiratory Rx at 36 wks PMA:** ☐ **None** (else, circle BOTH O2 & Vent support below:)
   O2 >21% ☐ Y / N ☐ NC≤2L +/- ☐ [0 **Vent** support: HFNC>2L$_1$ / N/(HF)CPAP$_2$ / NIMV$_3$ / Conv MV$_4$ / HFV$_5$]

2. Discharge/Death **weight** : ___________ g ☐ Discharge **Head Circ** ____.___cm

3. Enteral **feeding** at discharge (or, ≤24 hr before death): ☐None, ☐HM only, ☐Formula only, ☐HM+(F or HMF)

4. On **day of** D/C, transfer, or death: (Any) **monitor** _0_ / ☐Yes **O₂** _0_ / ☐Yes **Vent support** 0 / ☐ [HFNC/ NCPAP/ NIMV/ CMV/ HFV]

(5., **if transferred) Reason for transfer** from AMC to other Hospital: __Growth/DC planning __Diagnostics __Surgery __Chronic Care __Other

|jmbp|nicudb.23|rev221223

PLEASE RETURN TO NICU OFFICE (B4), Mail Code 101, ext 25421)
DO NOT FILE WITH MEDICAL RECORD!!

2023 ALBANY MEDICAL CENTER **2023** label on reverse side, please -->

NEONATOLOGY QA/QI DATABASE ***DO NOT FILE WITH MEDICAL RECORD- Keep in NICU!!***

Name: MOE ✓M __F _U; 'A', 'B', 'C'... **(Check circle in ☐; O if Outside Dx, Rx)**

⇨ 0 **RESPIRATORY Diagnoses:**

☐RDS ☐TTN ☐BPD ☑MAS ☑; O PTX Other [______ PulmHemorrh Pneumonia (or, LRTI)]

⇨ 0 **RESPIRATORY Support:**

[☑ N/(HF)CPAP ☐ +Nasal IMV(any)] ☐ + HFNC > 2L (=CPAP) ☐ + NC ≤ 2L (=flow)

☑IMV, ☐HFV, date off 7/18 ; 1.1=(no date if **only** for **OR+** <3days) ☑Surfactant (≤1500 g only) __ hrs __ min @ 1st dose

☑ O2, date off 7/20 Surf via: ☑ETT ☐LMA ☐Thin cath ☐Aerosol

☐ Caffeine(any) ☐Vit A IM ☐; **O** Steroids, systemic (RespRx) start date __/__ ☐; **O** iNO

⇨ 0 **CARDIOVASCULAR:**

☐PDA (not incidental) ☐ Indomethacin(1)/ ☐ Ibuprofen(2) / ☐Acetam.(4) ☐; **O** Ligation/Plug ☐PPHN

☐OTHER CV [(hypotension), hypertension, CHD (specify): hypovolemia] ☐Hydrocortisone start __/__

⇨ 0 **RENAL/FLUID/ELECTROLYTE:**

☐Renal failure ☐Other 'lyte problem: [______ LoNa<130 HiNa>150 RTA≤15 HiK LoGlycemia<40 HiGlycemia+Rx]

⇨ 0 ☐; **O** probiotics **NEC:** [☐; **O** Yes(1) ☐"NEC-like"(2) ☐; **O** SIP/focal perf only(3)] ☐Surgery for any of these (1)

(Dates): ☐ SIPclin Dx / ☐ SIPsurgDx (specify procedure below)

⇨ 0 **SEPSIS bacterial/fungal** (**Blood/CSF** culture **only**; specify organism(s)); or, Viral/"TORCH++":

☐ early ≤ 3 days: Organism ______ Viral ______

☐ late > 3 days: Organism(s)Episode1 ☐; **O** ______

Organism(s)Episodes2+ ☐; **O** ______

Presumed sepsis (Treated fully, but cultures negative) [☐early (1) ☐late(2) **(1&2 =3)**]

⇨ 0 **NEUROLOGIC:** ☐Seizures ☐Asphyxia/HIE ☐Hydrocephalus

HUS/CT/MRI: ☐0(None), or ☐IVH(0-4) [1__ 2__ late__ late__ __ __] ☐PVL: Y / N (all <1501 g w/ any imaging)

☐ [Other Neuro: ______ Perinatal depression ☐ **HypothermiaRx**]

⇨ **ROP exam:** ☐Yes / (No) Stage 0-5; (Zone) [__;(__) __;(__) __;(__) __;(__) __;(__) __;(__)] ☐; **O** Laser/Cryotherapy

(Dates): ☐; **O** Anti-VEGF drug

⇨ 0 **Other SURGICAL PROCEDURES (w/Gen or spinal anesth):** (PDA, ROP, above; **specify NEC surgeries) (+Location** ☐; **O**)

\+ ☐; **O** Hernia repair -- ☐; **O** ______oscopy +☐; **O** Dx or Rx Catheterization ______ ☐; **O** Fetal Surg

OTHER Procedure(s) 1 ☐; **O** ______

OTHER Procedure(s) 2 ☐; **O** ______ ☐ **+Surg Site Infection**

OTHER Procedure(s) 3 ☐; **O** ______

⇨ 0 **MAJOR MALFORMATIONS:** (Additional, not noted above; include TTTS, metabolic dis.)

Syndrome, or describe: ______

⇨ 0 **OTHER MAJOR NON-INCIDENTAL DIAGNOSES/causes of** (continued) **hospitalization:** (circle or print)

Adrenal insufficiency Apnea++ Chemical rickets>600 Cholestasis(DB>2) Drug exposure GER HiBili++ HiBili+Breast-FJ Hypothermia<35 (IDM) MRSA colonization NAS Neutropenia<1500 Polycythemia Poor po feeding SGA Suck-swallow incoordination Thrombopenia

1. LGA
2.
3. Anemia at birth, nuchal cord
4.
5.
6.

⇨ Notes (not to be entered into DB):

UVC position: (Low) / OK / high

STUDIES: HIE NIRS Provia

Morbidities/QA: TPN infiltrate: skin / liver Nasal septal breakdown — *Dates*

DRsurf_Brady_at ___ mL

...Unplanned Extubation dates: 7/18 ___ ___ ___

☐

☐

☐

☐ Follow-up Dx, Path/Genetics______

2023 **Albany Medical Center, NICU QI Database** **2023**

(Place **Label here**, or write)

Last Name: DOE *Sex:* __M ✓F __U *If multiple birth* (A) B C D

Medical record #: 7777777

DOE Female

Date of: Birth (MM/DD/YY) 07/14/23 *Admission Date:* ___/___/___

Ethnicity+ Race (mother): __ *Hispanic?* + __*Black* ✓*White* __*Asian* __*Native American* __*Pac Isl* __*Other*

Birth Location: ☐*Inborn* ☐*Outborn at* OSH via ER *Hospital* +(VON) **AMC adm for:** ✓Med Rx/Diagnostics __CoolingRx __Surgery __Chronic Care __Growth/DC planning __Other

Birthweight: 1410 *grams* *Gestational age:* 31 *weeks* *Apgars* 6 1/ 8 5 (__10) _

(=9999 for NICU readmissions; complete only first 3 lines above)

⇨ ☐ Admitting Attending (circle): JBC RC MFay MF GG MH RK MMB UM (JP) KT

****

ADMITTING ATTENDING VERIFY ABOVE & COMPLETE ADMISSION SECTION:

Delivery: ☑*Vaginal* ☐*C/Section*

⇨ **0 DR INTERVENTION(s):** ☑O2 ☑CPAP ☑Bag-mask/PPV ☐NasalIPPV ☐LMA ☐ETT(any) ☐Epi ☐Compressions

If ≤ 1500 grams *OR* ≤ 29 6/7 weeks, liveborn (VON criteria), enter these 9 items:

Prenatal Care (1 office visit): ☑Yes / No ☐ **DM$_{any}$** Yes / (No) ☐ **Chorioamnionitis** Yes/ (No) ☐ **Hypertension (any)** (Yes) / No ☐ **MagSO4** (Yes) / No

☐ **Best EGA** 31 weeks 5 **days** **Antenatal steroids:** __☐None ☐Incomplete ☑Complete (>1 - <7 days)

☐Birth **Head Circ** 30.0 cm ☐ **First AMC Temperature** 36.3 °C ☐Within 1 hour ☑At >1 hour of life

************************* **DISPOSITION / DISCHARGE** *************************

Chart to: Cerone Chowdhry Fay Fisher Geis Horgan Khalak (Monaco-Brown) Munshi Pinheiro Tauber

If died, *Date: (MM/DD/YY):* ___/___/___ **DR death?** _0_ /☐Yes **Autopsy** _0_ /☐Yes **inNICU<12 hrs?** _0_ /☐Yes

(*If DR death, note also: Surfactant, Malformations, on reverse side*)

If discharged, *date: (MM/DD/YY):* 8/2/23

To: ☐*Home (or foster care)* ☐*E6 Nursery/C7/D7* ☐*PICU* ☑*Other Hospital/(Name)* OSH

ALL DIAGNOSES & RXs FINALIZED? Check small print items **& boxes for VLBW/VON babies…**

If ≤1500 g BW ***or*** ≤29 weeks (VON): Date when 36 wks PMA: ___/___/___ ((N/A if D/Cd))

1. **Respiratory Rx at 36 wks PMA:** ☐ **None** (else, circle BOTH O2 & Vent support below:)
   O2 >21% ☐ Y / N ☐ NC≤2L +/- ☐ [0 **Vent** support: HFNC>2L$_1$ / N/(HF)CPAP$_2$ / NIMV$_3$ / Conv MV$_4$ / HFV$_5$]
2. Discharge/Death **weight**: 1780 g ☐ Discharge **Head Circ** 32.5 cm
3. Enteral **feeding** at discharge (or, ≤24 hr before death): ☐None, ☐HM only, ☐Formula only, ☑HM+(F or HMF)
4. On **day of** D/C, transfer, or death: (Any) **monitor** _0_ / ☑Yes **O₂** (_0_) / ☐Yes **Vent support** (0) / ☐ [HFNC/ NCPAP/ NIMV/ CMV/ HFV]

(5., **if transferred**) **Reason for transfer** from AMC to other Hospital: ✓Growth/DC planning __Diagnostics __Surgery __Chronic Care __Other

|jmbp|nicudb.23|rev221223

PLEASE RETURN TO NICU OFFICE (B4), Mail Code 101, ext 25421)

DO NOT FILE WITH MEDICAL RECORD!!

2023 ALBANY MEDICAL CENTER **2023** label on reverse side, please -->

NEONATOLOGY QA/QI DATABASE ***DO NOT FILE WITH MEDICAL RECORD- Keep in NICU!!***

Name: DOE __M ✓F _U; ('A') 'B', 'C'... (Check circle in ☐; O if Outside Dx, Rx)

⇨ 0 **RESPIRATORY Diagnoses:**
☑RDS ☐TTN ☐BPD ☐MAS ☐; O PTX Other [________ PulmHemorrh Pneumonia (or, LRTI)]

⇨ 0 **RESPIRATORY Support:**
[☑ N/(HF)CPAP ☐ +Nasal IMV(any)] ☑+ HFNC > 2L (=CPAP) ☐ + NC ≤ 2L (=flow)
☐IMV, ☐HFV, date off ___/___ ; 1.1=(no date if **only** for **OR+** <3days) ☑Surfactant (≤1500 g only) 1 hrs 14 min @ 1st dose
☑O$_2$, date off 7/16 Surf via: ☐ETT ☑LMA ☐Thin cath ☐Aerosol
☑Caffeine$_{any}$ ☐Vit A_IM ☐; O Steroids, systemic (RespRx) start date ___/___ ☐; O iNO

⇨ 0 **CARDIOVASCULAR:**
☐PDA (not incidental) ☐ Indomethacin(1)/ ☐ Ibuprofen(2) / ☐Acetam.(4) ☐; O Ligation/Plug ☐PPHN
☐OTHER CV [hypotension, hypertension, CHD (specify):________] ☐Hydrocortisone start ___/___

⇨ 0 **RENAL/FLUID/ELECTROLYTE:**
☐Renal failure ☐Other 'lyte problem: [________ LoNa<130 HiNa>150 RTA≤15 HiK LoGlycemia<40 HiGlycemia+Rx]

⇨ 0 ☐; O probiotics **NEC:** [☐; O Yes(1) ☐"NEC-like"(2) ☐; O SIP/focal perf only(3)] ☐Surgery for any of these (1)
(Dates): ☐ SIPclin Dx / ☐ SIPsurgDx (specify procedure below)

⇨ 0 **SEPSIS bacterial/fungal** (**Blood/CSF** culture **only**; specify organism(s)); or, Viral/"TORCH++":
☐ early ≤ **3 days:** Organism ________ Viral ________
☐ late > **3 days:** Organism(s)Episode1 ☐; O ________
Organism(s)Episodes2+ ☐; O ________
Presumed sepsis (Treated fully, but cultures negative) [☐early (1) ☐late(2) **(1&2 =3)**]

⇨ 0 **NEUROLOGIC:** ☐Seizures ☐Asphyxia/HIE ☐Hydrocephalus
HUS/CT/MRI: ☐0(None), or ☐IVH(0-4) [1 0 2 0 late __ late __ __ __] ☐PVL: Y / (N) (all <1501 g w/ any imaging)
☐ [Other Neuro: ________ Perinatal depression ☐ **HypothermiaRx**]

⇨ **ROP exam:** ☐Yes / (No) Stage 0-5; (Zone) [__;(_) __;(_) __;(_) __;(_) __;(_) __;(_)] ☐; O Laser/Cryotherapy
(Dates): ☐; O Anti-VEGF drug

⇨ 0 **Other SURGICAL PROCEDURES (w/Gen or spinal anesth):** (PDA, ROP, above; **specify NEC surgeries) (+Location** ☐; O)
+ ☐; O Hernia repair -- ☐; O ________oscopy +☐; O Dx or Rx Catheterization ________ ☐; O Fetal Surg
OTHER Procedure(s) 1 ☐; O ________
OTHER Procedure(s) 2 ☐; O ________ ☐+**Surg Site Infection**
OTHER Procedure(s) 3 ☐; O ________

⇨ 0 **MAJOR MALFORMATIONS:** (Additional, not noted above; include TTTS, metabolic dis.)
Syndrome, or describe: ________

⇨ 0 **OTHER MAJOR NON-INCIDENTAL DIAGNOSES/causes of** (continued) **hospitalization:** (circle or print)
Adrenal insufficiency Apnea++ Chemical rickets>600 Cholestasis(DB>2) Drug exposure GER HiBili++ HiBili+Breast-FJ Hypothermia<35
IDM MRSA colonization NAS Neutropenia<1500 Polycythemia Poor po feeding SGA Suck-swallow incoordination Thrombopenia
1. Breech, hip laxity 4.
2. 5.
3. 6.

⇨ Notes (not to be entered into DB):

UVC position: Low / OK / (high) ↑ O

Metab. screen CAH (+)

STUDIES: HIE NIRS Provia

Morbidities/QA: TPN infiltrate: skin / liver Nasal septal breakdown
DRsurf_Brady_at ____ mL
...Unplanned Extubation dates: ____ ____ ____ ____ ____
☐
☐
☐
☐ Follow-up Dx, Path/Genetics________

Dates

Supplementary Materials S2

Resources and basic functions needed for implementation of the database system

Functional steps	Resource	Alternate or accessory resource	Comments
Database development and maintenance	Excel® user with moderately advanced skills		Must be capable of using features for data validation, writing formulas, conditional formatting, generating and editing macros, creating advanced filters and queries.
Data gathering	Pink paper form		Cheap, visible, accessible in seconds at point of care
	Neonatologists	Other attending physicians	Record validated data during care episodes< Supervising physician, ideally.
Data check #1<br>(completion and basic logic checks)	Administrative assistant	Clerk or data registrar	Located in NICU, permits daily, timely interaction with service neonatologists. Administrative assistant, clerk and data registrar can be the same person
Data entry in local database<br>(+data check #2)	Administrative assistant	Clerk or data registrar	Logic checks and validation built into electronic worksheet<br>Selected cross-validation with information in discharge summaries
	Computer running Excel®	Other spreadsheet or database program	Both data sheets, computer and database file(s) must be appropriately secured
Data check #3<br>(cross-validation)	Database manager (neonatal clinician)	Neonatologist or another neonatal clinician	Use "Pink Sheet Police" (see Methods) and/or other small lists or datasets (e.g., ROP list) for cross-checking and further corrections
Data entry in external databases	Data registrar	Clerk, administrative assistant	Selected data entry into other databases e.g., VON, New York State (SPDS)
Data analyses	Data analyst	Neonatologist	Analyses done with or by a neonatologist.<br>Final cross-checks with other databases, annual cohorts.
	Purpose-built Excel® workbooks	Statistics programs, e.g., Stata®, SPSS®	Excel workbooks customized for specific analyses, with data transformation formulas, macros, graphics, tables.<br>Statistics programs for further automated analyses using purpose-written code

The individual who develops the electronic database should be facile with Excel® or another database program and be capable of customizing the database application or troubleshooting as needed. This individual need not be a clinician but should collaborate closely with a clinician to develop the data collection and coding system in the most efficient and effective manner possible. As neonatologists enter new data during daily work, no measurable added time is required. An administrative assistant (or clerk, or data registrar) is needed for data and workflow management, ensuring timely completion of the sheets and data entry; they should be familiar with vocabulary and common NICU processes, to maximize correct entries in free text fields and to detect obvious omissions. Having this individual located in the NICU or neonatology office area greatly facilitates the tagging and immediate completion of incomplete pink sheets by neonatologists, after patient discharges. Using this workflow, the person-time required for pink sheet completion and data entry is minimal, when forms are complete. According to administrative assistants who have entered data over the years, more time is required to follow up on incomplete sheets than on other database tasks. The database manager should be a neonatal clinician capable of reconciling inconsistencies and ultimately ensuring good data quality. Data submission to external databases may rely on the administrative assistant or other data entry staff.

A data analyst is fundamental. In our case, the same individual (JP) performs the developer and analyst roles. As noted in Methods, some data analysis functions are built into worksheets of the working file. Analysis of annual cohorts is augmented by other Excel-based tools with customized formulas, macros, and graphs; in addition, repeated analyses are automated through scripts developed in additional statistical software such as Stata or SPSS. Whereas automated analyses are important for consistency and efficiency, we cannot overemphasize the value of cross-checks and partly manual analyses performed by (or with) a clinician who is familiar with the plausibility of results. Annually, we also detect erroneous values by comparing reports from the pink sheet database, the VON Nightingale reporting system, SPDS, or limited administrative or research datasets available ad hoc. We commonly find data errors in each of these systems and correct them as possible, or alert those who supplied alternate data.

Supplementary Figure S1: Automated graph displaying NICU daily census, and average for the year-to-date census

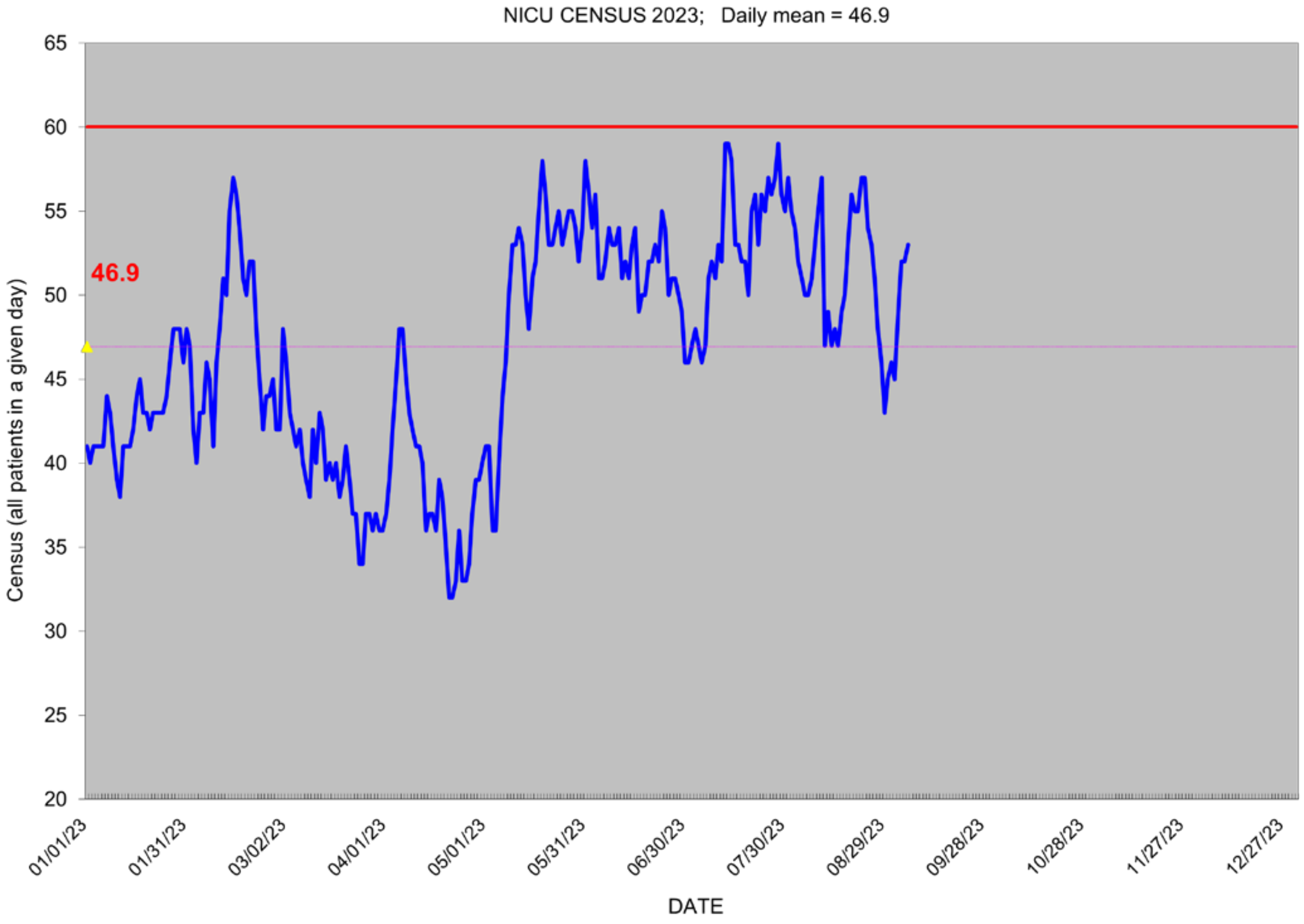

Supplement: Supplementary file 1 [file children-11-00217-s001.zip › children-2814868-supplementary.pdf]
